# Analysis of Load-Bearing Capacity and Lateral-Torsional Buckling (LTB) Stability of OSB Laminated and I-Beams Made of Wood and Wood-Based Materials

**DOI:** 10.3390/ma18235325

**Published:** 2025-11-26

**Authors:** Arkadiusz Denisiewicz, Tomasz Socha, Krzysztof Kula

**Affiliations:** Institute of Civil Engineering, University of Zielona, Góra ul. Prof. Z. Szafrana 1, 65-246 Zielona Góra, Poland; t.socha@ib.uz.zgora.pl (T.S.); k.kula@ib.uz.zgora.pl (K.K.)

**Keywords:** timber engineering, flexural performance, experimental studies, numerical analysis

## Abstract

The study presents experimental and numerical analyses comparing two types of wood-based structural beams—laminated beams made of oriented strand board (OSB) and traditional wooden I-beams. Load-bearing capacity, stiffness, and susceptibility to loss of stability were compared. Full-scale four-point bending tests were conducted on 5400 mm-long beams with 300 mm cross-sectional height, in accordance with the European Organisation for Technical Approvals (EOTA) guidelines. The results showed that OSB laminated beams, despite having about 20% lower bending stiffness, achieved up to 46% higher ultimate load capacity compared to I-beams. Failures in I-beams were mainly initiated by material defects and weaknesses in the glued joints of flanges, whereas laminated beams exhibited more uniform and predictable behaviour with greater repeatability of results. Numerical simulations performed in Abaqus/Standard 2022, including linear buckling and modal analyses, confirmed the experimental findings and indicated higher global stability and dynamic stiffness of laminated beams. The numerical model was validated in the elastic range and subsequently used to assess stability and dynamic behaviour, which are governed by linear elastic stiffness. The obtained results demonstrate that OSB laminated beams can serve as a potential alternative to conventional I-beams in structural applications requiring high safety and durability, offering a structurally efficient and more failure-tolerant configuration.

## 1. Introduction

Contemporary timber construction is developing dynamically, utilizing advanced wood processing technologies and modern wood-based composites, which enable the design and implementation of more efficient and durable structures [1,2]. Wood and wood-based materials play an increasingly important role in modern construction, aligning with the global trend of sustainable development. Their attractiveness stems not only from a low carbon footprint and renewable sourcing but also from a wide range of mechanical properties that can be tailored through appropriate engineering processes. Timber structures, ranging from traditional designs to modern mass timber systems, are being identified as forward-looking solutions capable of reducing CO_2_ emissions in the construction sector [3,4].

In recent decades, engineered wood products (EWPs), such as oriented strand board (OSB), laminated veneer lumber (LVL), laminated strand lumber (LSL), parallel strand lumber (PSL), cross-laminated timber (CLT), and glued laminated timber (GLT) have been intensively developed [5,6,7,8,9,10,11,12,13,14,15,16,17,18,19,20], allowing for rational use of wood raw materials, including low-quality timber and sawmill waste [21]. A particularly important group consists of strand-based EWPs, which, due to their high material yield and favourable mechanical properties, are widely used in structural elements, including the flanges and webs of I-beams [21]. In recent years, the importance of sandwich panels and wood-based composites has been growing, as they allow for increased material efficiency while maintaining high load-bearing capacity and stiffness. The authors of the study [22] point out that the development of wooden sandwich panels represents a key direction in wood materials engineering, enabling the optimization of mechanical properties while keeping the self-weight low. Meanwhile, the authors of the study [23] emphasize the importance of geometry and material parameters in honeycomb composite structures, which confirms the potential of such systems in modern timber construction. I-beams, in particular, are gaining popularity in floor and roof constructions [24,25], combining high stiffness with low self-weight. Their popularity also stems from material efficiency, low production costs, and ease of prefabrication, making them an attractive solution in both single-family and multi-family housing, and increasingly in public utility buildings.

In addition to wood and traditional wood-based materials, alternative lignocellulosic raw materials, such as bamboo, are also being used more frequently. Studies show that I-beams made from laminated bamboo can achieve high load-bearing capacity and stiffness, while also being a material with a low environmental footprint. However, their structural efficiency depends on the design of connectors and methods of reinforcing critical zones [26]. Similar development directions include attempts to use waste materials, e.g., recycled particleboards combined with wooden elements using mechanical connectors, which supports the idea of a circular economy [27].

Despite their many advantages, I-beams may exhibit significant limitations due to their material construction—primarily the presence of finger joints in flanges made of solid wood joined with micro-dowels [28], as well as the presence of natural wood defects such as knots, cracks, or variability in mechanical properties along the flanges. These defects can significantly weaken the element locally and reduce its load-bearing capacity, especially in the lower flanges subjected to tension [28]. As a result, in the presence of discontinuities or damage, I-beams may fail suddenly, reducing their reliability in applications requiring a high level of safety. In such cases, the catalogue load-bearing capacity declared by the manufacturer is not maintained. This poses a considerable problem for manufacturers, not only in terms of ensuring the required strength parameters of such products but also in terms of reputation. As a result of the research and analysis presented in this article, one of the world’s leading manufacturers of such products decided to withdraw from its production.

Various reinforcement strategies are proposed in the literature, including continuous or segmental reinforcement with carbon fibre reinforced polymer (CFRP) composite strips, which can not only increase load-bearing capacity but also mitigate the impact of local defects [25]. Research results indicate that appropriately selected reinforcements can increase the load-bearing capacity of beams by several dozen percent while simultaneously improving their deformation performance [28]. However, reinforcing I-beams is not always a rational solution. Reinforcements using composite materials, though effective, often lead to a significant increase in costs, complexity of the manufacturing technology, and challenges in ensuring the durability of adhesive or mechanical joints [25]. Moreover, in the case of elements with low initial load capacity, the application of additional reinforcements may not result in a proportional improvement in performance parameters relative to the incurred costs. Therefore, it becomes justified to seek alternative structural solutions that could compete with traditional I-beams. One such approach involves laminated beams, in which favourable mechanical properties can be achieved through appropriate material configuration while simplifying the production process.

This article attempts to answer the question of whether laminated beams made from OSB panels can serve as a functional alternative to I-beams in terms of their strength and reliability, while also identifying potential limitations of such a solution. The comparison of these two types of structural elements aimed to evaluate their behaviour under conditions close to real-world scenarios, including the effects of material defects and joint execution. The impact of wood defects and joint locations on the failure mechanisms of I-beams was also analyzed, with particular emphasis on the lower flanges, which are most exposed to tensile stresses.

As part of the study, a series of destructive four-point bending tests was conducted on beams with a span of 5400 mm and a section height of 300 mm, accompanied by an in-depth analysis of the obtained results. These tests were carried out in accordance with the guidelines of the European Organisation for Technical Approvals (EOTA), ensuring the quality and comparability of results.

The experimental studies were complemented by numerical simulations performed in the Abaqus/Standard 2022 [29] environment using a validated three-dimensional model, recreated based on the actual geometric and material parameters of the tested elements. These analyses included both buckling analysis to determine critical loads and modes of stability loss, as well as modal analysis to identify natural frequencies and vibration modes. The results of the numerical simulations were used to assess the global stability of the beams and compare their dynamic stiffness, providing a valuable complement to the experimental research and confirming the accuracy of the applied computational model.

## 2. Materials and Methods

In order to determine the differences in the behaviour of glued beams made of OSBs and BS-D 300 type I-beams, experimental bending tests were conducted. The tests were carried out in accordance with EOTA guidelines, using a four-point loading scheme that allows for the determination of the load–deflection relationship up to the point of failure.

The selection of two different cross-section types was motivated by the need for a detailed comparison between traditionally used I-beams and a new concept in the form of glued beams. I-beams, due to their construction consisting of a thin-walled web and solid flanges, are characterized by high stiffness with relatively low self-weight, which makes them a popular solution in lightweight structural systems. However, a potential drawback is their sensitivity to the quality of flange joint execution and the presence of weakened zones, which can determine the load-bearing capacity of the entire element.

In contrast, glued beams, produced by bonding layers of OSBs, do not have weak points in the form of flange joints and feature a more uniform material structure. As a result, they may demonstrate greater repeatability of test results, predictability of behaviour, and higher load-bearing capacity under ultimate conditions.

The comparison of these two types of beams allows for the identification of potential directions for optimizing timber structures, where stiffness and load-bearing capacity, as well as ensuring safe use of the structure, play a key role.

**Figure 1:**
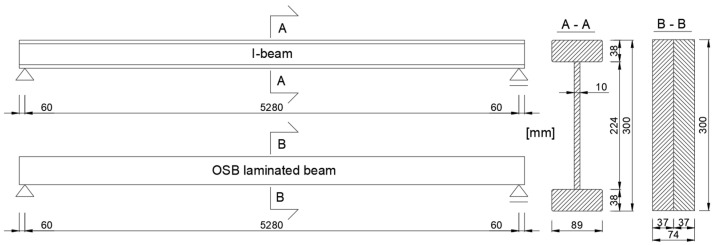
Geometry of the tested beams.

For research purposes, two groups of structural elements were used: the first group consisted of three double-layered laminated OSB beams, while the second group included three BS-D 300 type I-beams. The geometry of the beams is shown in Figure 1.

The laminated beams were made from EUROSTRAND OSB in a two-layer configuration, bonded with a two-component polyurethane adhesive (2K PUR), cured at room temperature under a pressure of 0.8 MPa for 12 h. The joints between the OSB web and wooden flanges in the I-beams were made using an industrial thermoplastic polyurethane adhesive (PUR). The bonding process was carried out at a temperature of 120 °C under a pressure of 0.6 MPa for 5 min. All specimens were conditioned for at least four weeks under laboratory conditions (20 ± 2 °C, relative humidity 55 ± 5%) before testing.

The mechanical properties of all materials used in the study are summarized in Table 1: E—longitudinal modulus of elasticity; E_t_—transverse modulus of elasticity; G—shear modulus; ν—Poisson’s ratio; MOR—bending strength. The values were obtained based on manufacturer data and verified in accordance with EN 300 [30] and EN 338 [31] standards.

Laboratory tests included:

- Preparation of OSB laminated beam samples and I-beam samples with identical geometric dimensions,
- Conditioning of samples under constant humidity and temperature conditions (20 ± 2 °C, 55 ± 5% RH),
- Execution of four-point bending tests in accordance with EOTA guidelines,
- Recording of force, displacements, and strains during loading,
- Determination of stiffness, ultimate load-bearing capacity, and failure modes of the tested beams,
- Analysis of the influence of material type (OSB, solid wood) and bonding technology on the repeatability of results,
- Photographic and descriptive documentation of the test process and failure mechanisms.

Numerical analyses included:

- Development of 3D spatial models of laminated beams and I-beams in the Abaqus/Standard 2022 environment,
- Assignment of actual material properties for OSB and solid wood,
- Validation of the numerical model in the elastic range based on laboratory test results,
- Execution of linear buckling analysis (Buckle Analysis) to determine buckling modes,
- Performance of modal analysis (Linear Frequency Extraction) to identify natural frequencies and mode shapes,
- Assessment of the influence of geometric and material parameters on the lateral-torsional buckling (LTB) stability of the tested elements.

### 2.1. Laboratory Tests

**Figure 2:**
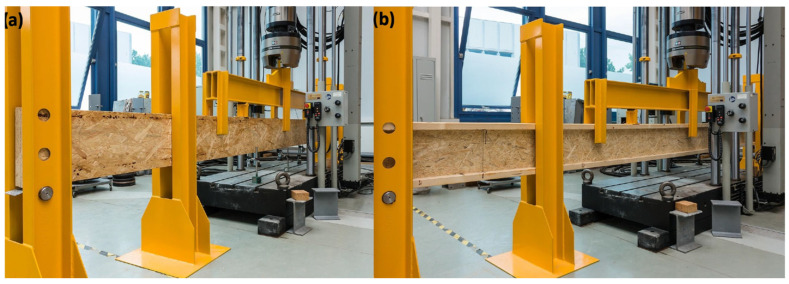
OSB laminated beam (**a**) and I-beam (**b**) on the test stand.

The research process consisted of two main stages: determining the stiffness and assessing the load-bearing capacity of the elements in a four-point bending test. Each element was tested under identical laboratory conditions, and all tests were conducted using an INSTRON 8804 testing machine (Instron Ltd., High Wycombe, UK) equipped with a 500 kN load cell (Figure 2). The force and mid-span deflection were continuously recorded using the Instron WaveMatrix control (Instron Ltd., High Wycombe, UK) and data acquisition system. Additional deflection measurements were carried out using external LVDT sensors from Novotechnik, Ostfildern, Germany (accuracy ± 0.01 mm).

**Figure 3:**
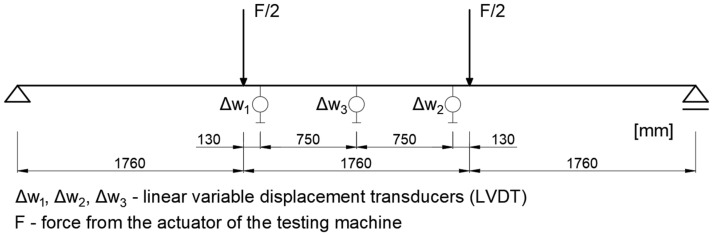
Static diagram of the tested system and deflection measurement setup.

In the first stage of the study, aimed at determining the stiffness of the tested beams, the samples were placed on supports with a span of 5280 mm and subjected to loading according to a four-point bending scheme, in which the pure bending zone covered a central section of 1760 mm–880 mm to each side from the centre of the beam (Figure 3).

Stiffness measurements were carried out using a two-stage loading process. Initially, each sample was loaded until the actuator displacement of the testing machine reached 10.0 mm, after which it was unloaded to a displacement of 2.0 mm. Following this preliminary phase, the main measurement phase began, involving reloading the beam with displacement increasing from 2.0 mm to 10.0 mm. During this phase, the mid-span deflection of the beam within the measuring setup was recorded with an accuracy of 0.01 mm, and the applied force was measured with an accuracy of 1.0 N. Based on the collected data, force-deflection graphs were created, which enabled the subsequent calculation of element stiffness. The bending stiffness was calculated using the following formula:

(1) $${\left(EJ\right)}_{beam}=\frac{∆F\cdot l\cdot l_{k}^{2}}{48\cdot ∆w_{4}}$$

where

${\left(EJ\right)}_{beam}$—bending stiffness [Nmm^2^],
∆F—increase in force during the change in actuator displacement of the testing machine from 2.0 mm to 10.0 mm [N],
l—span of the beam measured between the axes of the supports [mm],
$l_{k}$—span of the deflection measurement system within the pure bending zone [mm],
$∆w_{4}$—deflection at the midpoint of the deflection measurement system span [mm].

Deflections were measured in the pure bending zone using a system of three linear variable displacement transducers (LVDTs) positioned as shown in Figure 3. Two sensors (Δw_1_ and Δw_2_) were placed symmetrically near the loading points, while the third sensor (Δw_3_) was located at midspan. The local deflection Δw_4_ was determined according to formula (2) as the difference between the midspan deflection and the average of the readings from the two external sensors.

(2) $${∆w}_{4}={∆w}_{3}-\frac{{∆w}_{1}+{∆w}_{2}}{2}$$

Such a measurement setup allowed the elimination of shear deformation effects and ensured that the recorded deflection values corresponded exclusively to the bending deformations within the constant moment region. The deflection measurement scheme shown in Figure 3 provided high accuracy and repeatability of the results, forming the basis for determining stiffness and assessing the behaviour of the beams under bending.

In the second stage of the study, focused on determining the load-bearing capacity of the elements, the samples were first unloaded to an actuator displacement of 0.0 mm. The beams were then reloaded until complete failure occurred. The loading was applied at an actuator displacement rate of 0.1 mm/s, and the total testing time until failure was approximately 10 min. During this stage, detailed observations were made regarding the mode and location of beam failure, and the relationship between the applied force and the corresponding deflection of the sample was recorded. Based on the measurement results, the maximum failure force was determined, which enabled the calculation of the bending capacity of the tested elements. The bending capacity was calculated using the following formula:

(3) $$M_{u}=\frac{F_{u}\cdot l}{6}$$

where

$M_{u}$—bending capacity [kNm]
$F_{u}$—maximum force recorded during the test [kN],
l—span of the beam measured between the axes of the supports [m].

All conducted tests were thoroughly documented with photographs, which enabled a detailed analysis of the failure mechanism and characteristics of each element’s destruction process. The obtained results were subjected to detailed statistical analysis to assess the repeatability and reliability of the measurements. The described testing methodology allowed for an objective comparison of the strength and stiffness parameters of I-beams and OSB laminated beams, thereby making it possible to formulate practical recommendations regarding their further application as well as potential structural and technological improvements.

### 2.2. Numerical Model

To expand the experimental analysis and gain a better understanding of the behaviour of the tested elements under load, numerical models of the I-beams and OSB laminated beams were developed in the Abaqus/Standard 2022 environment [29]. These models reflected the actual geometry of the beams, with a clear span between supports of 5280 mm and a cross-section height of 300 mm. Their material parameters, boundary conditions, and loading scheme corresponded to the configuration used in the laboratory tests. A linear-elastic material behaviour and small displacements were assumed, which allowed for elastic state analysis and stability assessment under uniformly distributed loads. The objective of the numerical modelling was to accurately reproduce the elastic stiffness of the beams and, on this basis, to conduct stability-related analyses. Therefore, the validation of the finite element model was intentionally limited to the linear elastic range. Since both the buckling and modal analyses are inherently linear eigenvalue problems, a model that accurately reflects the elastic stiffness ensures reliable prediction of critical load factors and natural frequencies.

**Figure 4:**
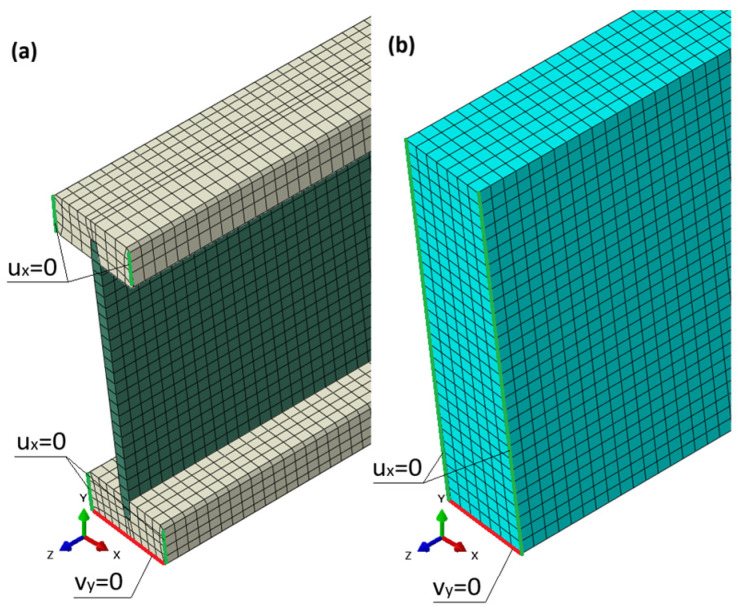
Numerical model of the I-beam (**a**) and the laminated beam (**b**).

Material parameters were selected based on manufacturer data: for pine wood, a Young’s modulus of E = 11,000 MPa, Poisson’s ratio ν = 0.20, and density ρ = 470 kg/m^3^ were assumed. For OSB panels, the values were E = 4930 MPa for the webs of the I-beams and 5430 MPa for the laminated beams, ν = 0.20, ρ = 650 kg/m^3^. The I-beam model reflected a classical structure composed of two wooden flanges and an OSB web, while the laminated beam model featured a solid OSB cross-section (Figure 4).

By adopting such a cross-section, the influence of the adhesive layer on the behaviour of the laminated beams was neglected. In the case of I-beams, a “Tie” connection [29] was used between the web and the upper and lower flanges. The adhesive layer between OSB laminations and between OSB and flange interfaces was not modelled explicitly. This simplification may lead to a moderate overestimation of global stiffness, but it does not significantly affect the comparative trends between beam types, as identical material assumptions were applied consistently in both models.

The models were developed in a three-dimensional form using eight-node finite elements of type C3D8R, which allowed for accurate representation of the stress state and local deformations of the cross-section. A finite element with a basic dimension of 10 mm × 10 mm × 10 mm was used. The I-beam model consists of a total of 111,041 finite elements, while the OSB laminated beam model has 58,608 elements. The support conditions of the beams in the model corresponded to those during the experimental tests. Figure 4 shows the method of applying boundary conditions. Horizontal displacements were constrained on edges marked in green, while vertical displacements were constrained on edges marked in red. During validation, displacements and force values from the model were read from the reference point RP, which was located at the centre of the span of the tested system, corresponding to the displacement readings from the actuator of the testing machine. The geometric dimensions of the model matched the dimensions of the beams tested experimentally, as shown in Figure 1. The beam length in the model was 5280 mm, which corresponded to the support point spacing during the tests.

In the buckling analysis, the Linear Buckling procedure in the Abaqus/Standard 2022 environment [29] was used, which leads to the solution of an eigenvalue problem for an elastic system in the finite element formulation [32]:

(4) $$\left(K+\lambda K_{g}\right)\phi =0$$

where

K—classical material stiffness matrix,
$K_{g}$—geometric stiffness matrix,
λ—eigenvalue (reference load multiplier),
ϕ—eigenvector (buckling mode shape).

For the assumed reference load *q_ref_* the critical load satisfies *q_cr_* = *λ_i_ q_ref_*, *i* = 1,2,3…. The smallest positive eigenvalue *λ_1_* gives the first (most critical) buckling mode. The values *λ* are also referred to as critical load factors, and their corresponding modes represent the buckling shapes of the structure. This analysis enabled the identification of the dominant instability mechanisms—both local and global—in both I-beams and laminated beams.

Since eigenvalue buckling analysis operates entirely within the linear-elastic domain and relies solely on the elastic stiffness matrix, its correctness is directly dependent on the accuracy of the elastic-range model validation. The experimentally confirmed agreement in the stiffness region provides the necessary basis to confidently apply the model to assess elastic stability modes and critical load factors.

Unlike the experimental studies, the dynamic analyses assumed fixed-end boundary conditions for all beams, as this support scheme is most commonly encountered in real operating conditions. For each model, the first six buckling modes were determined, which allowed for the assessment of their spatial stiffness and sensitivity to torsional-flexural deformations (Figure 15 and Figure 16).

The second stage of the numerical calculations involved a modal analysis of the Linear Frequency Extraction type, in which the natural frequencies and their corresponding vibration modes were determined based on the solution of the system’s eigenvalue equations. This is one of the fundamental tools of dynamic analysis, used to assess the stiffness of the system, the risk of resonance, and the localization of potential zones of dynamic strain concentration. In the finite element formulation, the dynamic system can be described by the matrix form of the equation of motion [33]:

(5) $$M\ddot{u}\left(t\right)+C\dot{u}\left(t\right)+Ku(t)=F(t)$$

where

M—mass matrix,
C—damping matrix,
K—structural stiffness matrix,
u(t)—displacement vector,
F(t)—external force vector.

In the analysis of natural vibrations, the effects of damping and external loading are neglected, i.e., ***C***= **0**, ***F***(*t*) *=* **0**, and the solution is sought in harmonic form:

(6) $$u\left(t\right)=\phi sin(\omega t)$$

where

ϕ—mode shape vector (eigenmode),
ω—angular frequency of natural vibrations [rad/s].

Substituting into the equation of motion leads to the classical eigenvalue problem:

(7) $$\left(K-\omega^{2}M\right)\phi =0$$

This is a second-order eigenvalue problem, whose solution consists of pairs (*ω_i_, Φ_i_*), representing the natural frequencies and corresponding mode shapes of the structure. This type of analysis enabled the assessment of the influence of geometry and material on the dynamic behaviour of the beams, as well as a comparison of the dynamic stiffness between the I-beam structures and the OSB laminated beams (Figure 17 and Figure 18).

Modal analysis also belongs to the class of linear eigenvalue problems and, similarly to buckling analysis, depends exclusively on the elastic stiffness and mass matrix. For this reason, experimental dynamic testing was not performed; instead, the stiffness-validated numerical model was used to extract natural frequencies and vibration modes. This approach is consistent with standard engineering practice for full-scale timber elements.

Although the main objective of this study is the experimental evaluation of full-scale laminated OSB and wooden I-beams, the numerical model plays a complementary and equally important role. In large-scale bending tests, it is difficult to precisely observe and quantify all the mechanisms of stability loss and failure, especially those involving local, torsional, or lateral–torsional buckling that may occur internally or out of plane. The validated finite element model developed in Abaqus/Standard 2022 therefore serves as a diagnostic and interpretative tool that allows for the detailed identification of such instability modes and stress redistribution phenomena, which cannot be captured through direct observation or conventional instrumentation.

The novelty of the numerical analysis lies in its application to reveal hidden instability mechanisms and evaluate the beam’s susceptibility to dynamic effects rather than in introducing a new computational technique. By combining the experimentally verified stiffness parameters with a geometrically faithful 3D representation, the model makes it possible to predict potential buckling modes, identify critical deformation regions, and assess the global stability and dynamic sensitivity of both beam types. This approach provides a valuable extension of the experimental findings and contributes to a more comprehensive understanding of the structural behaviour of lightweight timber and OSB laminated beams.

## 3. Results and Discussion

The obtained experimental and numerical results allowed for a comprehensive evaluation of the mechanical performance and stability of both analyzed beam types. The discussion presented in this section focuses on the comparative analysis of load–deflection relationships, ultimate bending capacity, stiffness, and observed failure modes. Special attention was given to identifying the influence of material composition and connection quality on the overall behaviour of the beams under bending. Additionally, the outcomes of numerical simulations were used to interpret the experimental observations and to assess the susceptibility of the beams to lateral–torsional buckling. It should be emphasized that the numerical model was not intended to replicate the nonlinear load-bearing capacity or post-elastic behaviour of the beams. Instead, its purpose was to explain experimentally unobservable instability mechanisms by means of elastic buckling analysis and to evaluate dynamic stiffness via modal analysis. The experimentally validated elastic stiffness ensures that such analyses yield physically meaningful and structurally consistent results. The correlation between laboratory tests and computational results provided a consistent basis for understanding the structural efficiency and reliability of OSB laminated beams compared to traditional wooden I-beams.

### 3.1. Results of Laboratory Tests

**Figure 5:**
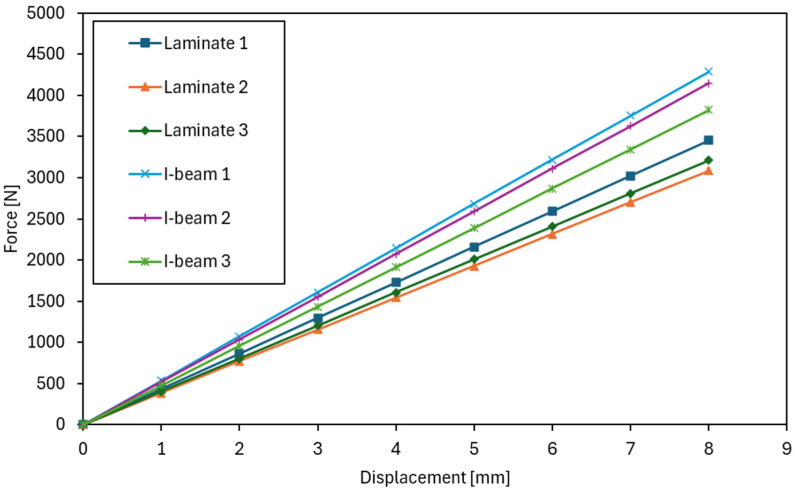
Force–displacement relationship during the stiffness test.

The laboratory tests began with determining the flexural stiffness of the tested beams. In the graphs showing stiffness in the elastic range (Figure 5), a clearly steeper slope is visible for the curves corresponding to the I-beams, which unequivocally confirms their higher flexural stiffness and smaller deflections under comparable loads. The influence of the web type on stiffness and the contribution of shear deformations was also observed in review studies of prefabricated I-beams: a reduction in the shear modulus of the web (e.g., in OSB panels vs. plywood/waferboard) increases the shear deformation component and may differentiate the slope of the F–u curves in the elastic range, which is consistent with our observations [24]. In contrast, the laminated beams were characterized by a gentler slope of the lines, indicating lower EJ values. However, their performance in this range was more uniform and predictable, with only minor differences between individual samples.

**Figure 6:**
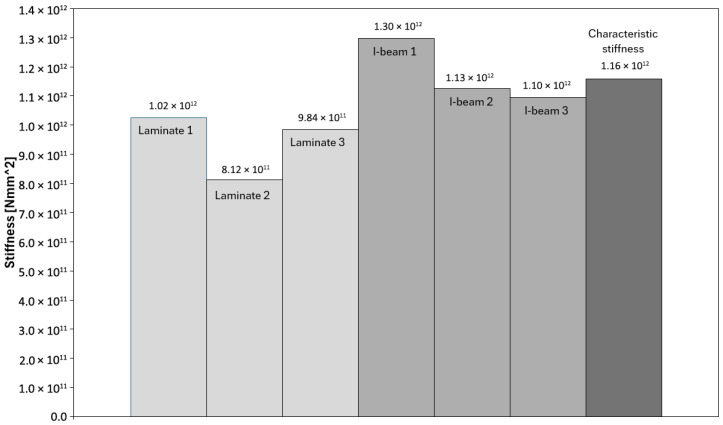
Stiffness of the tested beams.

In the tests of the I-beams, an average flexural stiffness EJ of 1.17 × 10^12^ Nmm^2^ was obtained, confirming the high resistance of these elements to deflection within the elastic range. Individual values for the samples (Figure 6) ranged from EJ = 1.10 × 10^12^ Nmm^2^ to EJ = 1.30 × 10^12^ Nmm^2^, indicating low variability. Sample I-beam 1 showed slightly higher stiffness than the others. For the laminated beams bonded from OSB, the average *EJ* value was 9.40 × 10^11^ Nmm^2^, with results ranging from 8.12 × 10^11^ Nmm^2^ to 1.02 × 10^12^ Nmm^2^. In this case, sample Laminate 2 exhibited lower stiffness compared to the others, which influenced the spread of values. Overall, however, the variation in stiffness values was similar to that observed in the I-beams.

**Figure 7:**
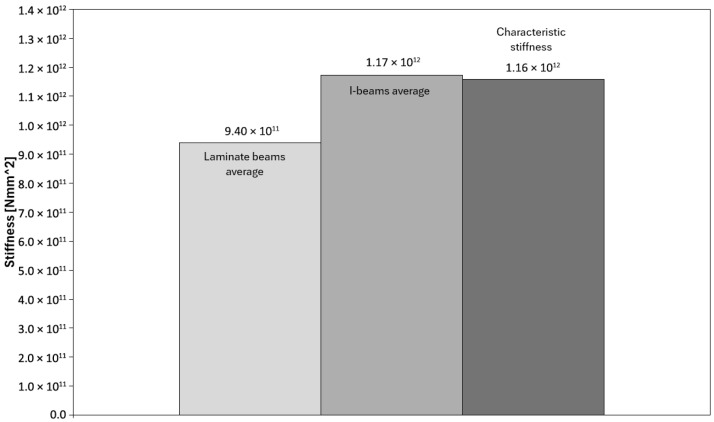
Average stiffness values of the tested beams.

Figure 7 presents a comparison of the average flexural stiffness values EJ for the tested laminated and I-beams, along with a reference to the characteristic value. In this study, the term “characteristic value” refers to the catalogue value provided by the beam manufacturer, corresponding to the characteristic bending strength determined in accordance with standards EN 408 [34] and EN 14080 [35]. The analysis shows that the I-beams achieved an average stiffness of 1.17 × 10^12^ Nmm^2^, which is very close to the assumed characteristic stiffness of 1.16 × 10^12^ Nmm^2^. This indicates that the test results align well with catalogue data and that the I-beams perform according to design assumptions in terms of stiffness. The laminated beams reached an average stiffness of 9.40 × 10^11^ Nmm^2^, which is approximately 20% lower compared to the I-beams. Despite this difference, these values are still sufficient to ensure proper structural performance and do not significantly deviate from expectations.

However, it should be noted that compared to the characteristic stiffness value, the laminated beams showed a clearly lower level, which confirms that their main advantage lies primarily in load-bearing capacity and behavioural consistency, rather than stiffness. Similar differences in bending stiffness between solid-section beams and I-beams were also reported in other studies [8,9,25], where webs made of OSB or LVL showed 10–25% lower stiffness compared to reference I-type beams. This was attributed to the lower shear modulus of the web material and the presence of adhesive layers. These findings are consistent with the observations obtained in the present study.

**Figure 8:**
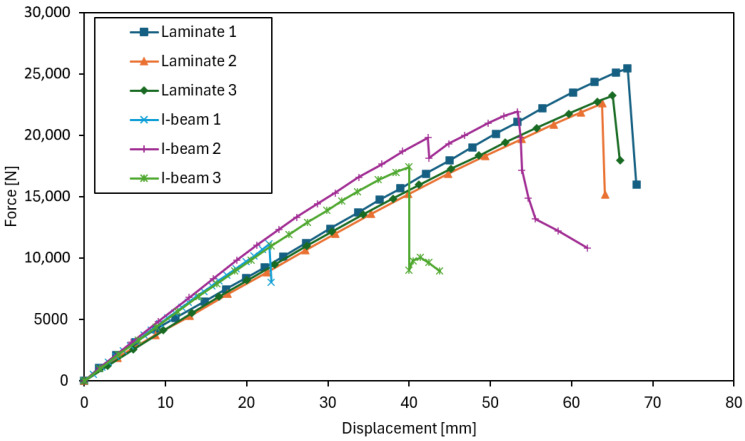
Force–displacement relationship during the load-bearing capacity test.

**Figure 9:**
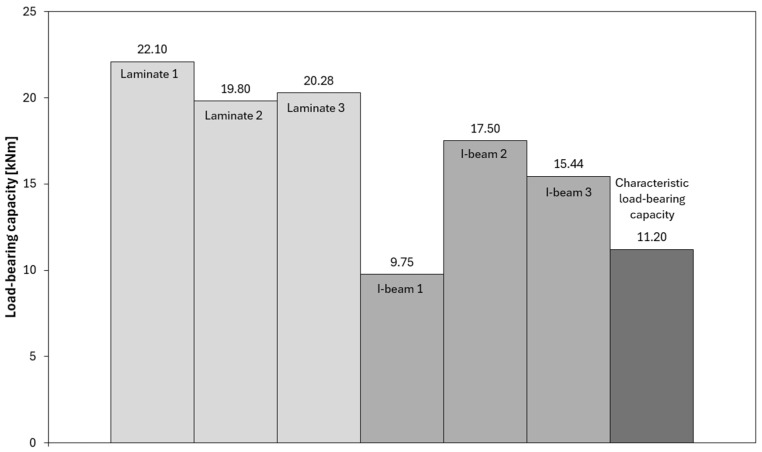
Load-bearing capacities of the tested beams.

**Figure 10:**
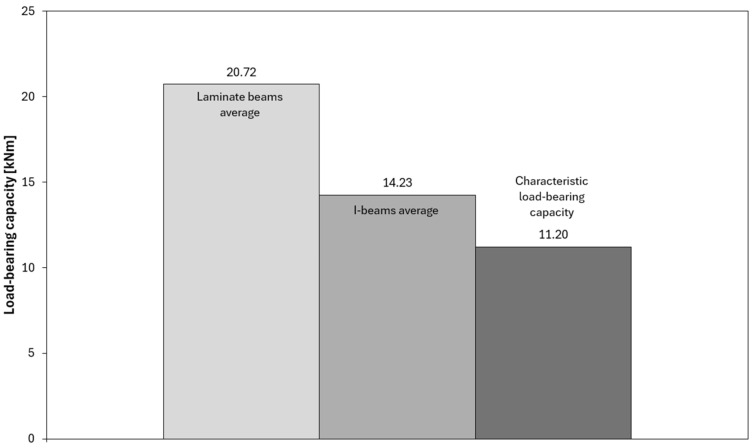
Average load-bearing capacity values of the tested beams.

The interpretation of the force–deflection graphs allows for deeper insights from the tests and provides a broader perspective on the behaviour of both analyzed beam types. In the graphs illustrating load-bearing capacity (Figure 8, Figure 9 and Figure 10), a clear increase in force is observed up to the point of reaching maximum values, followed by sudden failure of the element. The laminated beams are characterized by significantly higher maximum load-bearing capacity, reaching values in the range of 20–22 kNm, with failure occurring at greater displacements compared to the I-beams. This indicates a higher capacity to carry ultimate loads and a more ductile behaviour. In the case of the I-beams, failure was more gradual and signalled in advance. During the tests, early weakening was observed in the area of the lower flange connections or supports, indicating the susceptibility of this type of cross-section to local material defects and structural weak points. Similar phenomena were observed in studies on composite wooden and bamboo beams, where elements with solid or laminated webs exhibited higher load-bearing capacity and more ductile behaviour compared to conventional I-beams with slender OSB webs [25,26,27]. The authors of these studies also noted that in traditional I-beams, damage was initiated by shear cracks in the web or delamination in the flange–web joint area, which is consistent with the observations made in the present study.

The analysis of the force–deflection curves (Figure 8) shows that both types of beams exhibit behaviour typical of elastic–brittle materials, without a distinct transition between elastic and post-elastic phases. The curves remain nearly linear until failure, followed by a sudden loss of load-carrying capacity once the maximum stress is reached. This characteristic response is consistent with the brittle nature of wood-based composites, where the rupture of the material or adhesive joints governs the failure process. Similar elastic–brittle behaviour, with an almost linear force–deflection relationship up to sudden failure associated with web cracking or flange–web joint delamination, was observed in experimental studies of lightweight wooden I-beams [25]. The authors emphasized that the absence of a distinct plastic phase is typical for wood-based composites, in which the failure process is primarily governed by brittle fracture of fibres or adhesive joints.

Therefore, it is difficult to define separate elastic and post-elastic stages for the tested beams. Instead, the discussion focuses on comparing stiffness and ultimate strength as indicators of material performance. The OSB laminated beams displayed a slightly greater ability to absorb energy before fracture, resulting in higher ultimate load values, while the I-beams showed premature cracking due to defects in the flange joints. This confirms that both beam types behave primarily in an elastic–brittle manner, but the laminated configuration provides a more uniform stress distribution and a more predictable global failure mechanism.

**Figure 11:**
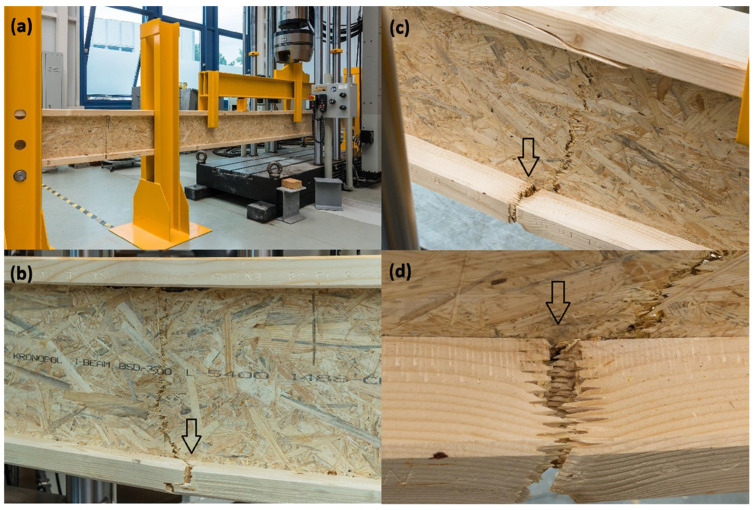
I-beam on the test stand (**a**), right and left sides of the beam after failure (**b**,**c**), damaged wedge joint of the bottom flange (**d**).

Among the tested I-beams, one sample (I-beam 1—Figure 9) showed a clearly deviating result, reaching a load-bearing capacity of only 9.75 kNm. This value was significantly lower than those of the other beams in this group and fell below the characteristic capacity, defined at 11.2 kNm. A detailed analysis revealed that the cause of the premature failure was a defective joint in the bottom flange, which acted as the initiation point of the damage process and ultimately limited the beam’s ability to carry loads (Figure 11).

Comparing the load-bearing capacities obtained in the tests with the characteristic capacity, it can be observed that most of the I-beams fell within a range close to or slightly above the reference value. Only the previously mentioned sample I-beam 1, with a capacity of 9.75 kNm, significantly lowered the average, confirming the sensitivity of this type of cross-section to local material and manufacturing defects. This result highlights the importance of quality and precision in executing the flange joints, which, in the case of I-beam elements, represent the weakest link of the entire structure (Figure 11).

In contrast, the laminated beams demonstrated not only higher load-bearing capacity compared to the characteristic values but also greater consistency in the results. All tested samples exceeded the reference capacity level, indicating stable and uniform material performance. Differences between individual beams were minimal, and the force–deflection curves showed repeatability and a predictable failure progression. This makes laminated beams a more reliable solution in both design and practical applications, especially in situations where ensuring a high level of safety and minimizing the risk of local, uncontrolled damage is essential.

**Figure 12:**
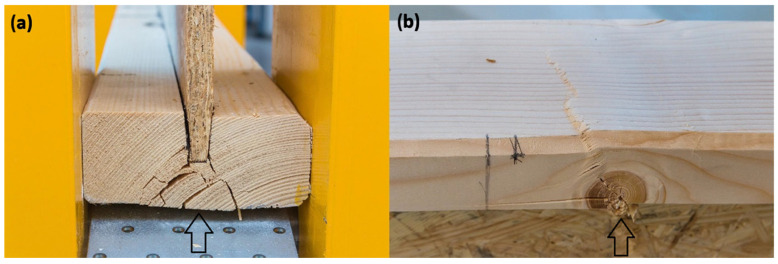
Damage to the flanges of I-beams in the support zone (**a**) and in the span (**b**).

Figure 12 presents examples of failure mechanisms observed in I-beams during laboratory testing. In Figure 12a, the initiation of failure in the region of the bottom flange of the I-beam is visible. Characteristic cracks radiate outward from the contact zone between the flange and the web, indicating a stress concentration at the joint. This type of damage is typical for I-beams, where the bottom flange operates in the tension zone, and any material defect or bonding imperfection can lead to failure initiation. A similar failure mechanism was described in study [27], indicating that in composite beams with a wood-based web, cracks were initiated in the tension zone at the junction of the flange and web, where stress concentrations occurred due to stiffness differences and imperfections in the adhesive joint. The authors emphasized that this area represents the weakest link in conventional I-section beams, and local delaminations or microcracks in this zone often trigger the brittle failure of the entire element.

Figure 12b shows damage to the top flange at the location of a natural wood defect in the form of a knot. The crack runs through the centre of the knot and propagates diagonally upward. This is an example of a situation where local material defects significantly reduce the element’s load-bearing capacity and may lead to premature failure of the beam.

The interpretation of both cases indicates that the main cause of reduced load-bearing capacity in I-beams lies in the weak points located in the flanges. These may result from both the quality of structural joints and natural material defects in the wood. These phenomena confirm that the flanges of such beams represent a critical area that determines their strength and reliability in structural performance.

**Figure 13:**
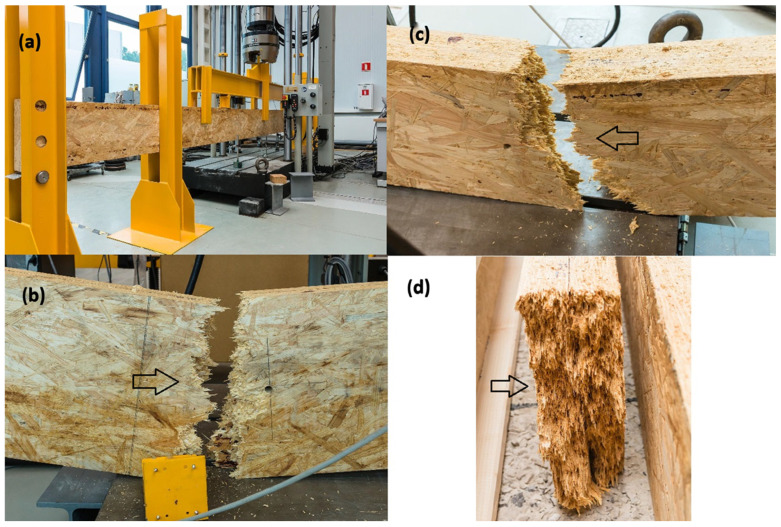
Laminated beam on the test stand (**a**), right and left sides of the beam after failure (**b**,**c**), cross-section view after failure (**d**).

Figure 13b,c show the laminated beam after failure—a characteristic split is visible in the central part, running through the entire height of the cross-section. This damage is extensive and continuous, involving both the tension and compression zones, which indicates a uniform stress distribution within the cross-section and the full engagement of the element up to the point of failure.

In other studies, failure in bamboo and OSB beams was attributed to the exhaustion of the strength of internal adhesive joints rather than to local defects, which confirms that laminated configurations exhibit more uniform stress fields and a predictable, cross-sectional failure mechanism [8,9]. Figure 13d presents a detailed view of the cross-section after failure, where delamination and OSB chip-out can be observed. This failure mechanism points to a dominant mode of damage caused by the exhaustion of internal bonding and material strength, without a clearly defined initiation point in a single localized area. The interpretation of this failure pattern leads to the conclusion that laminated beams do not have critical weak zones, such as joints or defects in the flanges found in I-beams. Their failure occurs in a more global and predictable manner, and the load-bearing capacities achieved during testing significantly exceeded characteristic values. As a result, laminated beams demonstrate greater reliability and behavioural consistency, which is a significant advantage in both design and practical applications.

### 3.2. Results of Numerical Simulations and Model Validation

The model was validated within the elastic range based on the results from experimental tests, and the boundary conditions as well as the loading scheme corresponded to the configuration used in the laboratory tests. The validation was not extended beyond the elastic range because the purpose of the numerical model was not to simulate nonlinear behaviour or failure, but to provide a reliable stiffness representation for subsequent modal and buckling analyses. These analyses, which are based exclusively on the structural stiffness and mass matrix, require accurate elastic properties rather than a full nonlinear material calibration.

To quantitatively compare the results of experimental tests and numerical simulations, an error summary was prepared for a mid-span deflection of u = 8 mm (Table 2). The table presents the force values corresponding to this displacement for individual beams and their numerical models, the absolute differences between the calculated results and the measurements, as well as the relative percentage errors. Additionally, the mean relative error was calculated for each group of beams, which allows for an assessment of the accuracy of the structural behaviour representation in the numerical model.

**Figure 14:**
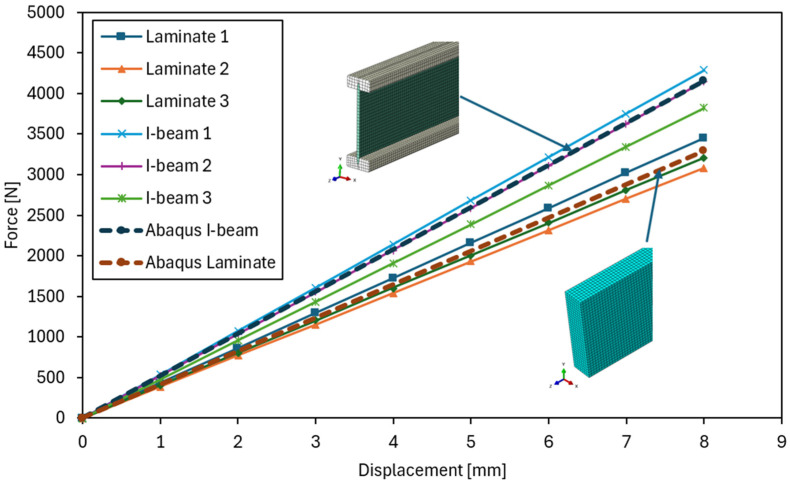
Numerical model validation.

Figure 14 presents the force–deflection relationship obtained from four-point bending tests for both laminated and I-beams, compared with the results of numerical analysis carried out in Abaqus/Standard 2022. The curves corresponding to the numerical models show very good agreement with the experimental results throughout the entire elastic range, confirming the accuracy of the adopted geometric, material, and boundary assumptions.

For both types of beams, the differences between the force values obtained from numerical analysis and laboratory tests did not exceed 5–8%, which can be considered very good agreement for this type of analysis. The obtained results confirm that the developed numerical model accurately reflects the behaviour of real structural elements and can be successfully used for further buckling analyses, allowing for the determination of critical load factors and buckling modes, as well as modal analyses, enabling the identification of natural frequencies and mode shapes of the tested elements. The purpose of these analyses was to assess the beam resistance to loss of stability and to determine its dynamic characteristics within the elastic range.

**Figure 15:**
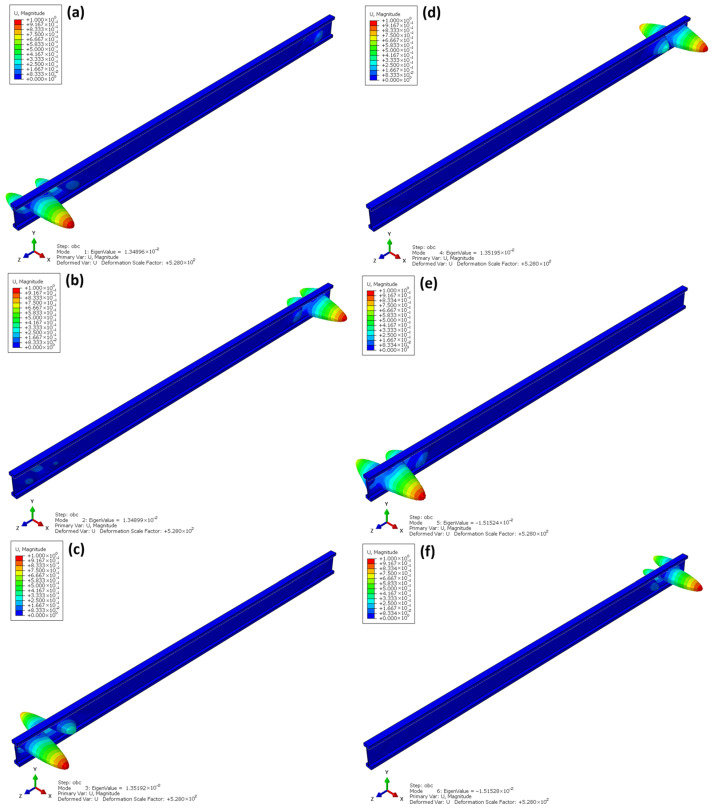
Linear buckling I-beam.

Figure 15 shows the first six buckling modes obtained from the linear buckling analysis (Linear Buckling) for the I-beams. The eigenvalues correspond to the critical load factors at which the structure loses elastic stability. As indicated by the analysis, the dominant buckling mode corresponds to local web instability under compression, which is typical for thin-walled beams with high web slenderness. The global torsional and lateral–torsional effects of the entire beam are present but relatively small compared to the pronounced web deformation and therefore are not clearly visible in the presented mode shape.

The first buckling mode (Figure 15a) appears at a very low eigenvalue λ = 1.35 × 10^−2^, which indicates that loss of stability may occur under a load corresponding to approximately 1.3% of the uniformly distributed reference load of 1 MPa applied to the top flange of the beam in the numerical analysis. This reference load represents the unit load condition adopted for the eigenvalue buckling computation. The deformations are concentrated in the support region, where twisting of the bottom flange and local web bulging are observed. The subsequent buckling modes (Figure 15b–f) exhibit a similar pattern—they are modes with an increasing number of deformation half-waves, in which flange twisting and web buckling occur alternately along the length of the beam.

Both the shape of the deformations and the very low values of the buckling factors indicate a high sensitivity of the structure to local and torsional–flexural buckling. This results directly from the geometry of the I-beam—the thin OSB web does not provide sufficient spatial stiffness, and the lack of lateral flange bracing intensifies torsional deformation. A similar local web buckling mechanism was noted in other numerical and experimental studies, where the thin-walled OSB web proved to be the critical element determining the loss of stability under compression [25]. These findings are consistent with the observations in the present study and confirm the need for additional stabilizing elements, such as sheathing or lateral bracing, which significantly increase buckling capacity and reduce torsional deformations [27].

**Figure 16:**
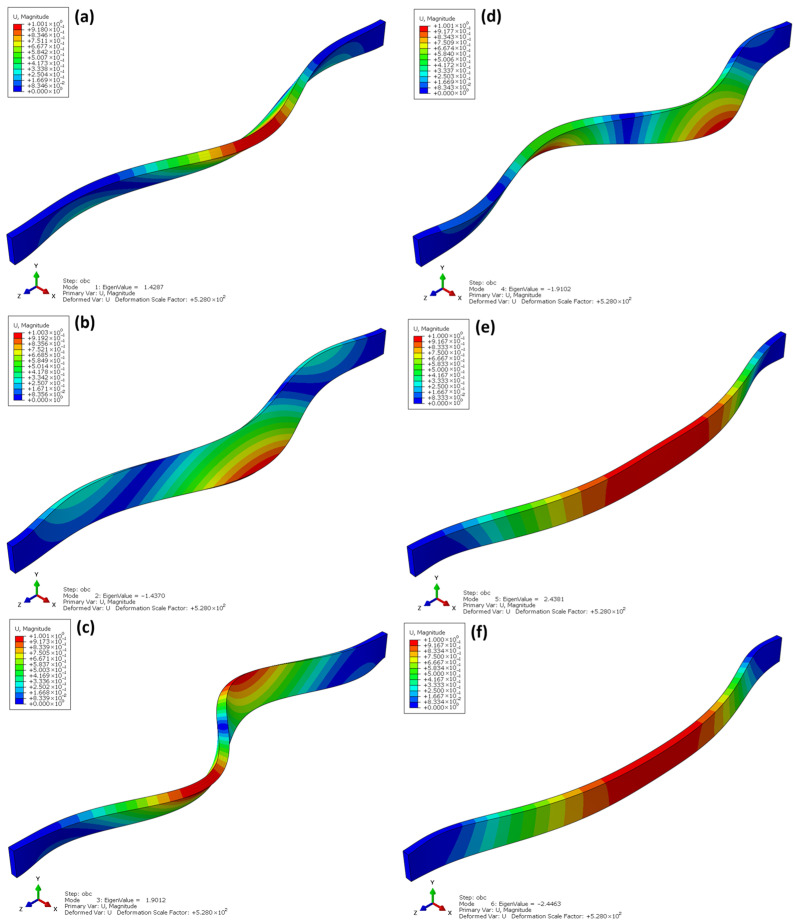
Linear buckling laminate beams.

Figure 16 shows the first six buckling modes obtained from the linear buckling analysis (Linear Buckling) for the laminated beams made of OSBs. The eigenvalues range from λ ≈ 1.43 to 2.45, indicating that the critical load at which stability is lost is over a hundred times higher than that of I-beams with comparable dimensions. This demonstrates the significantly greater stiffness and global resistance of the laminated beam structure.

The deformation shapes shown in Figure 16a–f indicate that the buckling of the laminated beams is global and flexural in nature, without local web deformations or cross-sectional twisting. In the first buckling mode (Figure 16a), a gentle deflection along the entire length of the beam is observed, typical for solid-web elements with a uniform stiffness distribution. In the subsequent modes (Figure 16b–f), the number of deformation half-waves increases, but the deformation remains symmetric, confirming the dominance of a global buckling mechanism.

The absence of local instabilities is a consequence of the continuous structure of the laminated OSB material, which ensures uniform stress transfer and eliminates stress concentration phenomena in joint areas—areas that do not exist in these beams. The high buckling factors confirm that laminated beams exhibit strong resistance to flexural buckling and have a favourable distribution of spatial stiffness, making them structurally more stable and predictable in service than traditional I-beams.

**Figure 17:**
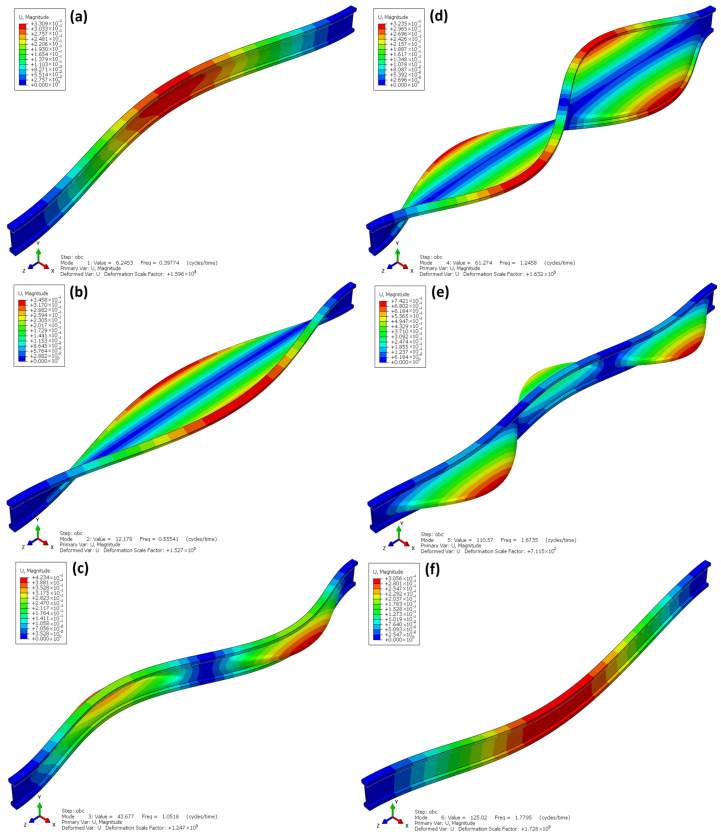
Linear frequency extraction I-beams.

Figure 17 presents the first six natural vibration modes obtained from a modal analysis of the Linear Frequency Extraction type conducted for the I-beam. The natural frequencies range from approximately 0.40 to 1.78 Hz, reflecting the combined effect of the beam’s stiffness and mass distribution rather than indicating its stiffness alone. The analysis identified both global modes, involving deflections along the entire length of the element (Figure 17a,c,f), and local modes, concentrated in the web and flange regions (Figure 17b,d,e). Similar coupled flexural–torsional vibrations were observed in studies of lightweight I-beams, in which the slender web and low torsional stiffness of the flanges determine the nature of the dynamic behaviour [25,27].

The first natural vibration mode (Figure 17a) corresponds to classical bending of the beam perpendicular to the plane of bending. The subsequent modes show an increasing number of half-waves and the appearance of complex torsional–flexural deformations. The third and fifth modes (Figure 17c,e) have a combined character—involving both bending and partial twisting of the cross-section, which is typical for beams with low torsional stiffness. In the modes where local deformations of the web are observed, they result from its low thickness and limited stiffness in the transverse direction.

The obtained results confirm that I-beams are characterized by a low fundamental frequency and a significant contribution of torsional behaviour in higher modes. This indicates a high susceptibility of the structure to resonance under low-frequency dynamic loading. The presence of local vibration modes also suggests the potential for dynamic instability phenomena to occur in the flange–web connection regions.

From an engineering standpoint, the modal analysis clearly confirms that I-beams require additional lateral bracing or the use of other elements that increase torsional stiffness in order to reduce their susceptibility to dynamic deformations.

**Figure 18:**
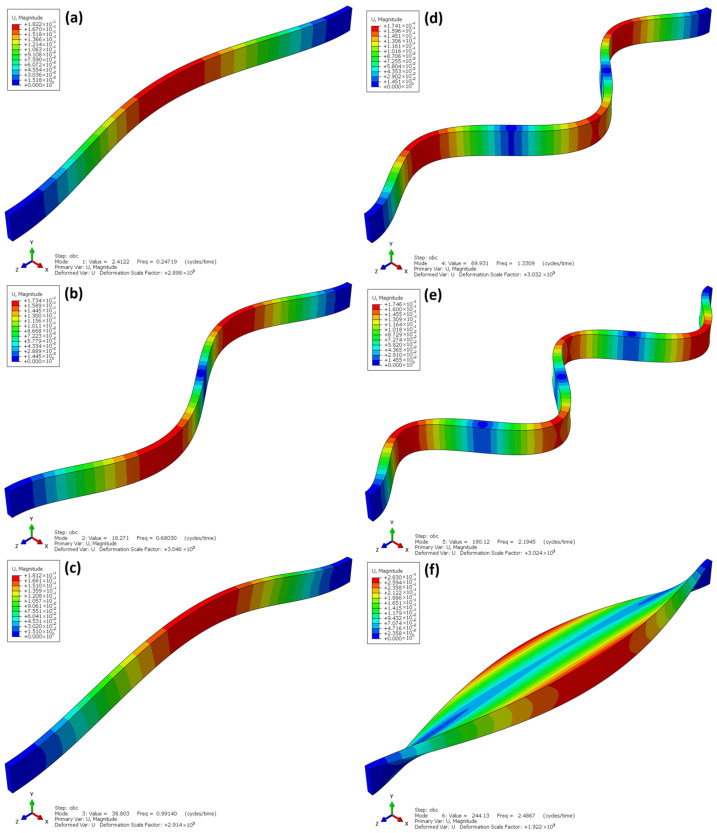
Linear frequency extraction of laminate beams.

Figure 18 presents the first six natural vibration modes obtained from a modal analysis of the Linear Frequency Extraction type for laminated beams made of OSBs. The natural frequencies range from approximately 0.25 to 2.49 Hz, indicating a dynamic stiffness comparable to that of the I-beams. The vibration modes shown in the figure clearly exhibit a global character—the deformation involves the entire length of the element, without local disturbances or cross-sectional twisting. Similar observations regarding the global nature of deformations without local disturbances were also confirmed in the literature [19]. Studies on glued laminated beams demonstrated that for a span-to-depth ratio of a/h > 3.0, the deformations are purely flexural, involving the entire length of the element, with no signs of local buckling or cross-sectional twisting. The authors emphasize that in such cases, the structural behaviour is global, and deformations develop continuously along the entire beam, which is consistent with the observations presented in the present study.

The first vibration mode (Figure 18a) corresponds to classical flexural bending in the main plane, with a smooth and symmetrical deformation pattern. The subsequent modes (Figure 18b–f) show vibrations with a higher number of half-waves, yet their shapes remain regular, without signs of local instability. Only at higher frequencies is a slight cross-sectional twist observed (Figure 18d,e), which is natural for long elements with a solid-web cross-section. The last mode shown (Figure 18f) has a global character, dominated by longitudinal bending, which is typical for beams with high geometric and material continuity.

The absence of local deformations and twisting—characteristic of I-beams—demonstrates that OSB laminated beams exhibit significantly higher dynamic stability and a more favourable distribution of spatial stiffness. The similar natural frequencies compared to I-beams suggest a comparable susceptibility to resonance under low-frequency excitations.

## 4. Conclusions

The experimental and numerical analyses carried out enabled a comprehensive comparison between OSB laminated beams and traditional wooden I-beams. The results confirmed that the laminated beams exhibit higher load-bearing capacity (up to 46% greater) and more predictable behaviour, despite having slightly lower bending stiffness (approximately 20% less). A uniform failure mechanism in the form of controlled cracking indicates a favourable stress distribution and the absence of weakened zones caused by joints or material defects.

The I-beams tended to fail prematurely in the bottom flanges due to the presence of defective finger joints or other discontinuities, highlighting their sensitivity to local defects.

The numerical model developed in the Abaqus/Standard 2022 environment accurately reflected the experimental results (with differences below 8%) and was used to assess stability and identify failure modes. The analyses showed significantly higher stability for OSB laminated beams, whose critical load factors exceeded those of I-beams by more than two orders of magnitude.

In summary, OSB laminated beams can be considered a sustainable and reliable alternative to conventional I-beams, offering greater resistance to local damage, better stability, and a simpler manufacturing process. These features make them a promising solution for timber structures that require a high level of safety and durability.

Future research should take into account long-term rheological effects and the impact of environmental conditions to more fully confirm their applicability in engineering practice.

## Figures and Tables

**Table 1:** Material parameters (manufacturer’s data).

Material	Density [kg/m^3^]	E [MPa]	E_t_ [MPa]	G [MPa]	ν [-]	MOR [MPa]
I-beam Pine C24 (flanges)	470	11,000	370	690	0.20	24
I-beam OSB (web)	650	4930	2100	1250	0.20	18
Laminated beam OSB	650	5430	2350	1320	0.20	22

**Table 2:** Error table comparing experimental and numerical results at mid-span deflection u = 8 mm.

Tested Element/Model	F [N]	Absolute Difference	Relative Error [%]	Mean Relative Error [%]
Laminate 1	3451	159	4.60	4.63
Laminate 2	3085	207	6.72	
Laminate 3	3209	83	2.58	
I-beam 1	4288	131	3.06	3.61
I-beam 2	4144	13	0.32	
I-beam 3	3869	288	7.45	
Abaqus Laminate	3292	-	-	-
Abaqus I-beam	4157	-	-	-

## Data Availability

The original contributions presented in this study are included in the article. Further inquiries can be directed to the corresponding author.
